# Mesenchymal stem cells for the adjunctive treatment of cytomegalovirus pneumonia in hematopoietic stem cell transplant recipients: a Single-center prospective study

**DOI:** 10.1007/s00277-025-06684-5

**Published:** 2025-10-08

**Authors:** Xianghui Liu, Xin Xia, Lingyun Bian, Zaihong Shen, Kun Zhou, Liping Wan, Xianmin Song, Yin Tong

**Affiliations:** 1 Department of Hematology, Shanghai General Hospital, Shanghai Jiao Tong University School of Medicine, Shanghai, 200080 China; 2 Department of Hematology, Taizhou First People’s Hospital, Huangyan Hospital of Wenzhou Medical University, Taizhou City, 318020 Zhejiang Province China

**Keywords:** Allogeneic hematopoietic stem cell transplantation, Cytomegalovirus pneumonia, Mesenchymal stem cells, Cell therapy

## Abstract

To evaluate the efficacy of mesenchymal stem cells (MSCs) as an adjuvant therapy for cytomegalovirus (CMV) pneumonia after allogeneic hematopoietic stem cell transplantation (allo-HSCT) through a prospective study. From June 2016 to June 2022, 43 patients were diagnosed with CMV pneumonia after allo-HSCT and received antiviral therapy, of whom 24 received MSCs infusion. The primary endpoints of the study were the cure rate and the time to cure of CMV pneumonia, and the secondary endpoint was the overall survival after CMV pneumonia. The 2-year overall survival post-pneumonia in MSCs group was significantly better than that in non-MSCs group (74% vs. 50%, *p* = 0.034). Cure rate of pneumonia in MSCs group was higher than that in non-MSCs group without statistical significance (79% vs. 63%, *p* = 0.245). Time (days) to cure in MSCs group was shorter than that in non-MSCs group without statistical significance (39[14–142] vs. 54[6-157], *p* = 0.765). The high mortality rate of CMV pneumonia after allo-HSCT leads to a poor prognosis. The use of MSCs improves the overall survival of CMV pneumonia. The cure rate and time to cure of pneumonia were not improved statistically. At present, there is still a lack of prospective studies on MSCs in the treatment of pneumonia after HSCT, which needs to be supplemented in the future.

## Introduction

Cytomegalovirus infection after allogeneic hematopoietic stem cell transplantation can lead to multiple organ diseases, among which CMV pneumonia is the most serious, and its mortality rate is reported to be 30–50% [1–5]. Although the adoption of pre-emptive antiviral therapy as the standard of care, along with the use of new prophylactic drugs, has led to a decrease in the incidence of post-transplantation CMV infections, CMV viremia remains a risk factor for non-relapse mortality after transplantation [6].

Mesenchymal stem cells (MSCs) possess immunomodulatory, antibacterial, and anti-inflammatory properties, and aid in lung tissue regeneration and remodeling, reduction of inflammation, and restoration of pulmonary fluid balance [7, 8]. MSCs can secrete several growth factors to promote the regeneration of type II alveolar epithelial cells, including vascular endothelial growth factor, hepatocyte growth factor, and keratinocyte growth factor [9]. At sites of inflammation, MSCs modulate the functions of immunocytes through direct contact and paracrine effects, involving effectors like prostaglandin E2, indoleamine 2,3-dioxygenase, human leukocyte antigen isoform, and transforming growth factor β [10]. Pulmonary lesions induced by influenza virus infection were reduced by MSCs treatment in both animal model and humans [11, 12]. Clinical trials have also demonstrated that MSCs are beneficial in improving the clinical, radiological, and immunological status of viral pneumonia [13–15]. More relevantly, they can increase the cure rate of severe pneumonia in children following hematopoietic stem cell transplantation [16].

This study analyzed the efficacy of MSCs for the adjunctive treatment of cytomegalovirus pneumonia in allo-HSCT recipients by comparing the clinical outcome of the MSCs group with that of the non-MSCs group, aiming to provide a reference for the treatment of CMV pneumonia, which has a high mortality rate.

## Patients and methods

### Patient eligibility

**Fig. 1:**
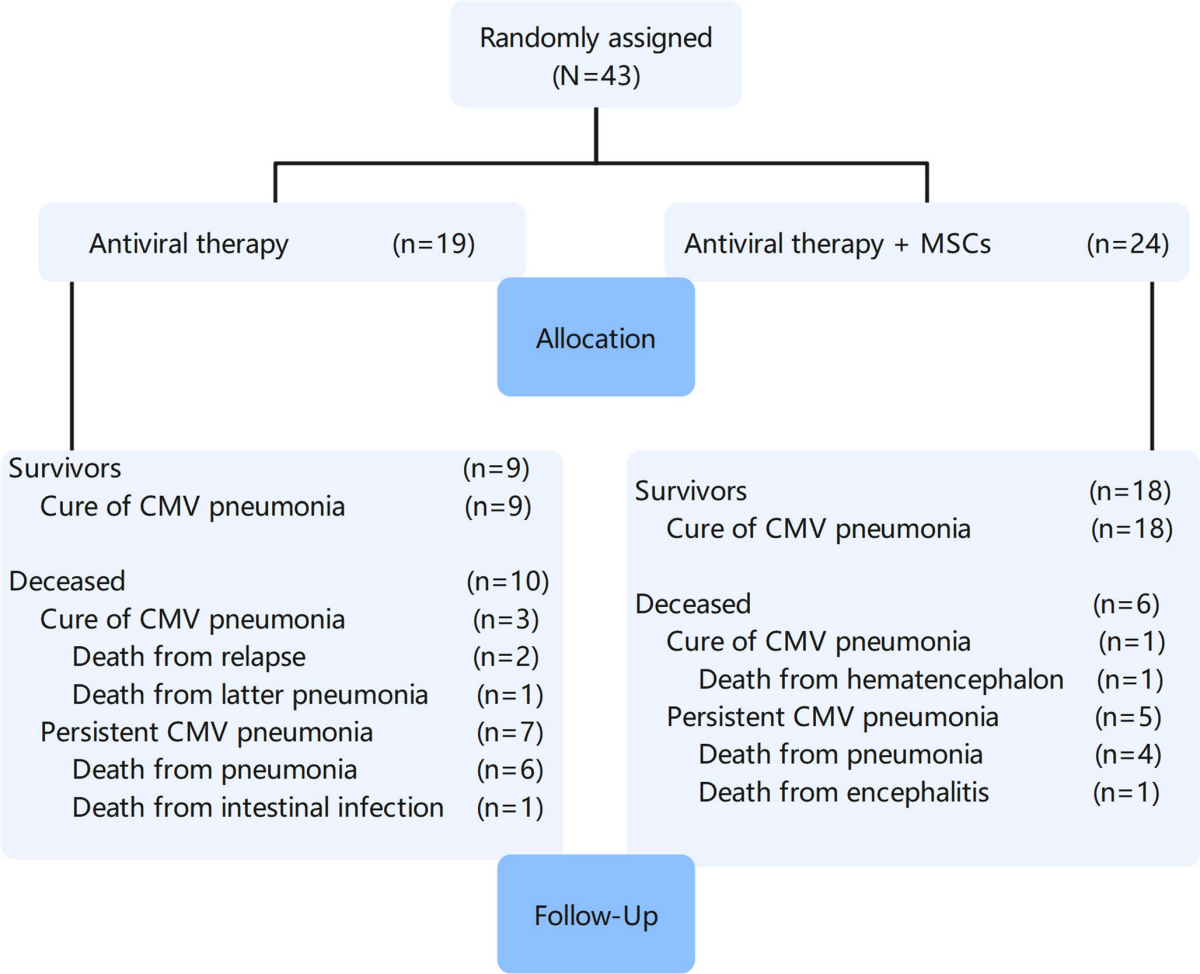
Patients allocation and follow-up

The study included 43 patients who underwent allo-HSCT in our department from June 2016 to June 2022 and were clinically diagnosed with CMV pneumonia through bronchoscopy. All patients received standard antiviral treatment, of which 24 patients were randomly administered mesenchymal stem cell therapy as an adjunct treatment (shown in Fig. 1). Simple randomization was performed using a random number table. There were no significant differences in baseline levels between the two groups, as described below. Exclusion criteria: presence of other severe complications at the time of CMV pneumonia diagnosis in the patient.

### Diagnostic criteria

In this study, the diagnosis of CMV pneumonia adheres to the diagnostic criteria proposed by the Cytomegalovirus Drug Development Forum in 2016 [17]. Proven CMV pneumonia: (i) Clinical evidence of pneumonia, such as new infiltrates on pulmonary imaging, shortness of breath, difficulty breathing, and other symptoms; (ii) Evidence of CMV in lung tissue biopsy specimens confirmed by viral isolation, rapid culture, histopathology, immunohistochemistry, or DNA hybridization techniques. Probable CMV pneumonia: (i) Clinical evidence of pneumonia; (ii) Evidence of CMV in bronchoalveolar lavage fluid, confirmed by viral isolation, rapid culture, or quantitative CMV DNA testing. In this study, no patients underwent lung biopsy, therefore, diagnosis were based on probable diagnostic criteria. As viral loads were detected with commercial assay different to the forum standard, the diagnostic threshold is not comparable to the guideline. Nevertheless, 95% of patients met a threshold of 1*10^7^ copies/L which supported the CMV infection [18]. The other two patients with lower BALF viral loads did not have any other pathogens detected and were considered to have CMV pneumonia based on radiographic findings. The date of pneumonia diagnosis refers to the date when new pulmonary infiltrates were identified on imaging.

### BALF assay techniques

Patients with pneumonia post-HSCT underwent bronchoalveolar lavage for definitive diagnosis. The procedural standards were based on the “Chinese Expert Consensus on Pathogen Detection in Bronchoalveolar Lavage Fluid for Pulmonary Infectious Diseases (2017 Edition),” and the assays included: exfoliative cytology, acid-fast staining smear, fungal smear, bacterial smear, CMV-DNA, Mycobacterium tuberculosis DNA, EBV-DNA PCR assays, bacterial and fungal cultures, with a subset of samples undergoing pathogen detection via Next Generation Sequencing (NGS) technology.

### Prevention and treatment of CMV infection

All patients were free of CMV viremia before transplantation. Prophylactic medication was administered to prevent CMV infection before and after transplantation: intravenous ganciclovir at 10 mg/(kg·d) (−7 ~ 1 d), followed by oral valganciclovir as viral prophylaxis after transplantation. For patients who experienced intolerable side effects such as decreased blood counts or impaired renal function, preventive medication was adjusted by switching to oral valacyclovir or acyclovir. Within the first six months post-transplantation, patients’ peripheral blood CMV-DNA was monitored weekly using the PCR method. For those diagnosed with CMV pneumonia post-transplantation, treatment included ganciclovir at a dose of 5 mg/kg every 12 h, or foscarnet sodium at 60 mg/kg every 8 h, along with intravenous immunoglobulin at 10 g/day for 3 days. In cases of hypoxemia or respiratory failure, methylprednisolone was administered at 1–2 mg/(kg·d). Chest CT scans were conducted weekly during hospitalization, with intervals extended for patients whose conditions stabilized, and monthly chest CT scans were conducted after discharge until the pulmonary inflammation was substantially resolved. As only three patients underwent transplantation after the approval of letermovir in China, none of the patients received letermovir due to limited drug accessibility and financial constraints.

### Prophylaxis and treatment of GVHD

For prophylaxis of GVHD, four types of regimens were utilized. For haploidentical donors, an ATG-based(10 mg/kg) or low-dose ATG/PTCy-based (ATG, 2.5 mg/kg/d on days − 2 to − 1; PTCy, 50 mg/kg/d on day 3) regimen was utilized. For matched sibling donors, the standard prophylaxis consisted of cyclosporine combined with mycophenolate mofetil and short-course methotrexate. For matched unrelated donors, a combination of cyclosporine, mycophenolate mofetil, methotrexate and ATG (5 mg/kg) was employed.

For Grade I acute GVHD, management involved local treatment and close observation. For Grade II or higher acute GVHD, first-line therapy consisted of systemic corticosteroids, with concomitant adjustment of the cyclosporine trough level to 150–250 µg/L. In cases of steroid-refractory or steroid-dependent disease, ruxolitinib was administered as second-line treatment.

### MSCs therapy

The MSCs used in this study were derived from umbilical cords, provided by Shandong Qilu Cell Therapy Engineering Technology Co., Ltd. Each MSCs batch was obtained from different donors, characterized by the surface markers CD90+, CD73+, CD105+, CD34-, CD11b-, CD19-, CD45-, and HLA-DR-. The cells met the following criteria: (1) absence of bacteria, fungi, or mycoplasma detected; (2) endotoxin levels ≤ 0.5EU/mL; (3) cell viability ≥ 90%. The dosage for the MSCs therapy group was 1 × 10^6^ cells/kg per infusion, administered once a week, with the total number of infusions ranging from 1 to 4 depending on the clinical outcome of the patient. Post-infusion, patients were monitored for any adverse reactions, including local redness, rash, darkened urine, limb pain, and any other reported discomforts.

### Data collection

The primary endpoints of this study are the cure rate of CMV pneumonia and the time to cure, while the secondary endpoint is the overall survival after diagnosis of CMV pneumonia. Cure of pneumonia is defined as significant improvement in both clinical symptoms and pulmonary radiographic findings. The time to cure is measured from the date of CMV pneumonia diagnosis to the date of cure. The overall survival post-pneumonia is calculated from the date of CMV pneumonia diagnosis to the date of death, or to the last follow-up date if the patient is alive or lost to follow-up. Data were collected through consultation of our hospital’s electronic medical record system and by telephone inquiries.

### Statistical methods

Quantitative data which follow a normal distribution (such as age) are described using mean ± standard deviation, while quantitative data not assumed to follow a normal distribution (such as the onset time of CMV pneumonia and time to cure) are described using median and range. Count data are presented as frequencies and proportions. Comparisons of quantitative data between the MSCs group and the non-MSCs group are conducted using the independent samples t-test (for normally distributed data) or the Mann–Whitney test (for non-normally distributed data). Comparisons of count data between the two groups are made using the chi-square test or Fisher’s exact test as directly obtained. Survival analysis is performed using the Kaplan-Meier method. Data processing in this study is conducted using SPSS 26.0.

## Results

### Patient characteristics

As shown in Tables 1 and 43 patients developed CMV pneumonia post-transplantation and received antiviral medication, of which 24 were treated with MSCs and 19 did not receive MSCs treatment. Comparison of baseline levels between the two groups revealed no significant differences, including age, gender, whether umbilical cord blood was transfused as an adjunct, donor-recipient matching, primary disease, disease remission status, and pre-treatment regimen.

**Table 1:** Baseline characteristics of post-HSCT CMV pneumonia patients

	MSCs group(*n* = 24) *n*(%)	Non-MSCs group(*n* = 19) *n*(%)	*P* value
Age(year)			
Mean ± SD	36 ± 13	36 ± 14	0.989
Gender			
male	10(42)	12(63)	
female	14(58)	7(37)	0.161
Unrelated UCB stem cell infusion			
Yes	7(29)	6(32)	
No	17(71)	13(68)	0.864
Donor type			
haploidentical	18(75)	11(58)	
syngeneic	0	2(11)	
unrelated	6(25)	6(31)	0.235
Stem cell source			
peripheral blood	24(100)	19(100)	/
Primary disease			
AML	11(46)	9(47)	
ALL	5(21)	4(21)	
MDS	3(13)	3(16)	
Lymphoma	1(4)	2(10)	
AA	4(16)	1(6)	0.903
Disease status			
CR	15(63)	14(74)	
PR	4(16)	1(5)	
non-remission	4(16)	3(16)	
relapse	1(5)	1(5)	0.437
CMV viremia pre-HSCT			
Yes	0	0	
No	24(100)	19(100)	/
Conditioning regimen			
myeloablative	21(88)	14(74)	
reduced-intensity	3(12)	5(26)	0.446
GVHD prophylaxis			
ATG-based	8(39.5)	4(39.5)	
low-dose ATG/PTCy	10(27.9)	7(27.9)	
CSA + MMF + MTX	6(32.6)	8(32.6)	0.452
aGVHD grade			
I	6(25)	6(32)	
II	2(8)	2(11)	
III	0	0	
IV	0	0	0.555
Type of aGVHD			
skin	8	8	
GI tract	0	1	
liver	1	0	0.250
MSCs sinfusion			
1	1(4)	/	
2	12(50)		
3	3(13)		
4	8(33)		/

Among the 43 patients, 29 cases (67%) were acute leukemia, including 20 cases of AML and 9 cases of ALL. Other diseases included MDS (6 cases), lymphoma (3 cases), and aplastic anemia (5 cases). There were 22 male patients (51%) and 21 female patients (49%). The average age of the patients was 36 ± 13 years, with 3 pediatric patients aged 8, 15, and 16 years respectively. The majority of the transplants (29 cases, 67%) were haploidentical related donor transplants, 12 patients received transplants from unrelated donors from a bone marrow registry (4 cases with 10/10 full match, 7 cases with 9/10 match, and 1 case with 8/10 match), and 2 cases received syngeneic transplant. Most patients (29 cases, 67%) were in complete remission of their primary disease at the time of transplant, 5 were in partial remission, and 9 were in relapse or non-remission. 81% of the patients received a myeloablative conditioning regimen before transplantation, while 8 patients underwent a reduced intensity conditioning regimen. All patients were infused with allogeneic hematopoietic stem cells from peripheral blood. None of the patients had cytomegalovirus viremia before transplantation.

Within 100 days post-transplantation, 16 patients (37%) developed acute graft-versus-host disease (GVHD), with 4 cases (2 in the MSCs group and 2 in the non-MSCs group) being grade II or higher. The GVHD primarily affected the skin and gastrointestinal tract, with the most severe reactions being grade III skin reactions in 2 patients. The median onset time for acute GVHD was 17 days post-transplantation (range, 2–58 days). There was no significant difference in the incidence of acute GVHD between the MSCs group and the non-MSCs group (*P* = 0.555).

The dosage of mesenchymal stem cells for each infusion was 1.0 × 10^6^/kg body weight, with an interval of 1 week between infusions. One patient (4%) received a single infusion, 12 patients (50%) received two infusions, 3 patients (13%) received three infusions, and 8 patients (33%) received four infusions. The median time to MSCs infusion was the 8th day (range, 2–22 days) following radiographic diagnosis of pneumonia.

### Clinical features of CMV pneumonia

#### Symptoms and radiographic findings

As shown in Table 2, the median onset time of CMV pneumonia in the MSCs group was 49 days post-transplantation (range, 35–139 days), slightly earlier than that in the non-MSCs group, which had a median onset of 59 days (range, 17–234 days; *p* = 0.172). The primary symptoms in the MSCs group were fever (18 cases, 75%), followed by cough and expectoration (12 cases, 50%), and chest tightness with shortness of breath (5 cases, 21%). Similarly, in the non-MSCs group, the symptoms were fever (14 cases, 74%), cough and expectoration (10 cases, 53%), and chest tightness with shortness of breath (5 cases, 26%). Radiographic changes in the lungs generally preceded clinical symptoms and displayed diverse manifestations, primarily including: (1) typical interstitial changes such as lobular central nodules and diffuse ground-glass opacities, (2) nodular changes with or without cavitation, (3) consolidation-like changes, (4) patchy infiltration. In the MSCs group, the various radiographic manifestations were observed in 12 cases (50%), 10 cases (42%), 3 cases (13%), and 6 cases (25%), respectively, while in the non-MSCs group, they were observed in 7 cases (37%), 7 cases (37%), 6 cases (32%), and 5 cases (26%), respectively.

#### BALF assay results

All 43 patients tested positive for CMV-DNA in their bronchoalveolar lavage fluid, with a median viral load of 7.2 × 10^7^ copies/L. All but two patients had a viral load over 1*10^7^ copies/L. The two patients with lower BALF viral loads did not have any other pathogens detected and were considered to have CMV pneumonia based on radiographic findings. The viral load values for both groups were converted to logarithms with base 10, and there was no significant difference between the groups (*P* = 0.058).

#### Co-infection

Based on the results of BALF assays, sputum smears and cultures, and radiographic examinations, 11 patients were diagnosed with co-infections. In the MSCs group, there were 5 cases of co-infection (1 case with fungal infection, 1 case with EBV, 1 case with Enterococcus, 1 case with Pneumocystis jirovecii, and 1 case with Mycoplasma). In the non-MSCs group, 6 cases of co-infection were identified (2 cases with fungal infections, 2 cases with Pneumocystis jirovecii, 1 case with Pseudomonas aeruginosa, and 1 case with Klebsiella pneumoniae), and all were treated with corresponding antibiotics.

#### CMV viremia

All 43 patients were tested negative for CMV-DNA in their blood before HSCT. At the time of CMV pneumonia diagnosis, 22 patients (92%) in the MSCs group developed CMV viremia, compared to 12 patients (63%) in the non-MSCs group (*P* = 0.057). The median time to onset of viremia in the MSCs group was 48 days post-transplant (range, 35–70 days), while in the non-MSCs group, it was 49 days post-transplant (range, 14–68 days), with no significant difference between the two groups (*P* = 0.403). At the diagnosis of pneumonia, 34 out of 43 patients were under antiviral treatment due to cytomegalovirus viremia, 7 were given prophylactic valganciclovir, and 2 were given oral valacyclovir prophylaxis.

The viral load of CMV-DNA in patients with viremia in the MSCs group ranged from 1.5 × 10^6^ to 5.4 × 10^8^ copies/L, and in the non-MSCs group, from 1.2 × 10^5^ to 1.6 × 10^9^ copies/L. The logarithmic values of viral loads between the two groups showed no significant difference (*P* = 0.333). Twenty patients (91%) in the MSCs group converted to CMV-DNA negative, with a median conversion time of 19 days (range, 3–77 days), compared to 9 patients (75%) in the non-MSCs group, with a median conversion time of 18 days (range, 4–75 days), showing no significant difference in conversion times between the two groups (*P* = 0.871). Five patients had persistent CMV viremia and prolonged pneumonia. They died at a median time of 100 days after diagnosis of pneumonia (range, 12–172 days).

In the MSCs group, 2 patients did not develop viremia during the CMV pneumonia episode but had been detected with viremia 35 days and 90 days before the diagnosis of this CMV pneumonia episode, respectively, and turned negative after antiviral treatment. In the non-MSCs group, 7 patients did not develop viremia during the episode, with 3 of them having been detected with viremia 22 days, 126 days, and 197 days before this CMV pneumonia diagnosis, respectively, and turned negative after treatment.

**Table 2:** Clinical features of CMV pneumonia (*n* = 43)

	MSCs group(*n* = 24) *n* (%)	Non-MSCs group(*n* = 19) *n* (%)	*P*
Median onset time post-HSCT, days (range)	49(35–139)	59(17–234)	0.172
Symptoms			
fever	18(75%)	14(74%)	1.000
cough	12(50%)	10(53%)	0.864
chest tightness	5(21%)	5(26%)	0.953
Co-infection			0.423
Yes	5(21%)	6(32%)	
No	19(79%)	13(68%)	
Viremia			0.057
Yes	22(92%)	12(63%)	
No	2(8%)	7(37%)	

### Adverse reactions

No acute infusion toxicity events, allergic reactions, or thrombotic/embolic events related to the infusion of MSCs were reported in the MSCs group.

### Cure rate, time to cure, and OS

By the end of follow-up, among 43 patients, 16 died. The median overall survival post-CMV pneumonia in deceased patients was 170 days (range, 12–843 days). The median follow-up time for survivors or lost to follow-up patients in the MSCs group was 1113 days, compared to 834 days in the non-MSCs group. Six patients in MSCs group died, 4 from pulmonary infection, 1 from hematencephalon and 1 from multiple organ failure and viral encephalitis. Ten patients in non-MSCs group died, 7 from pulmonary infection and 3 from relapse. The cure of CMV pneumonia was defined as significant improvement in both clinical symptoms and radiological findings, with the date of radiological improvement designated as the date of cure.

**Fig. 2:**
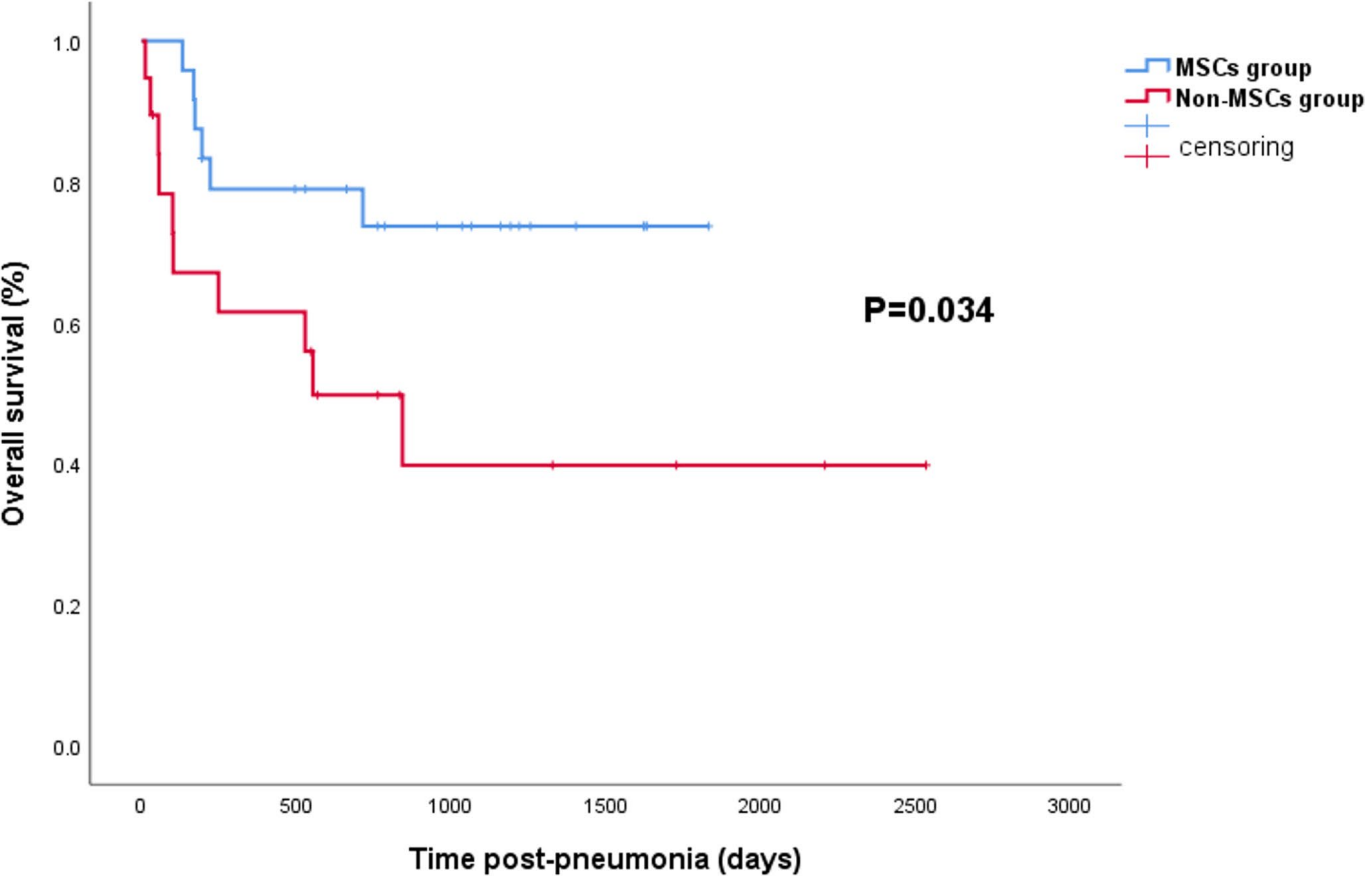
Survival curves of patients with CMV pneumonia in the MSCs group and non-MSCs group, *p* = 0.034

As shown in Table 3, the cure rates for CMV pneumonia in the MSCs group and the non-MSCs group were 79% and 63%, respectively, with a p-value of 0.245. The median time to cure CMV pneumonia was 39 days (range, 14–142 days) in the MSCs group and 54 days (range, 6–157 days) in non-MSCs group, showing no significant difference (*p* = 0.765). The survival curves following CMV pneumonia is depicted in Fig. 2. The 2-year overall survival rates were 74% in the MSCs group and 50% in non-MSCs group, indicating a significant difference between the two groups (*p* = 0.034).

**Table 3:** Efficacy and prognosis in CMV pneumonia patients

	MSCs group(*n* = 24) *n* (%)	Non-MSCs group(*n* = 19) *n* (%)	*P*
Cure			0.245
Yes	19(79%)	12(63%)	
No	5(21%)	7(37%)	
Time to cure, days (range)	39(14–142)	54(6–157)	0.765
Median time to viral clearance, days (range)	19(3–77)	18(4–75)	0.871
Two-year OS post-pneumonia	74%	50%	0.034

We also analyzed other potential factors that might affect the cure rate of CMV pneumonia, including gender, age, donor type, primary hematological disease, GVHD prophylaxis, whether in complete remission at the time of transplantation, viral load of CMV DNA during viremia, and concurrent infections with other pathogens. None of these factors were found to significantly impact the cure of CMV pneumonia (*P* > 0.05).

### Radiological residual lesions

Among the cured patients in both groups, a total of 31, one patient from each group lacked long-term radiological follow-up data post-cure. For the remaining 29 patients (18 for MSCs group and 11 for non-MSCs group), the median time of the last chest CT scan at follow-up after the diagnosis of CMV pneumonia was 357 days. In the MSCs group, 9 patients had residual pulmonary radiological changes, compared to 8 patients in non-MSCs group. The residual pulmonary radiological manifestations included: (1) pulmonary interstitial changes (radiological descriptions include interstitial thickening, widespread thickening of the small lobe septa); (2) scattered small nodular and patchy shadows, a few patchy opacities, scattered fibrous strands, and fibrocalcific foci; (3) atelectasis of the lower lobes, and cavitation.

## Discussion

In the early stages of the development of allogeneic hematopoietic stem cell technology, before the advent of CMV prophylactic therapies, the incidence of CMV pneumonia could reach 10–35% [19]. And the mortality rate approached 100% [20]. Although the incidence of CMV pneumonia has decreased to < 2% [21] with the standardization of viremia monitoring and preemptive treatment, and the use of new prophylactic drugs is expected to further reduce the incidence, the mortality rate remains high at 30–50% [1–4]. By the end of the follow-up in this study, 10 out of 43 patients died from CMV pneumonia, with a median survival time of 117 (12–248) day. This indicates that CMV pneumonia severely impacts allogeneic hematopoietic stem cell recipients.

MSCs have already been used in graft-versus-host disease after HSCT [22]. Moreover, current research on the adjunctive therapy of MSCs for post-transplantation pneumonia suggests their potential in improving patient outcomes [16]. In this study, the use of MSCs significantly improved patient survival, with a 2-year overall survival rate in the MSCs group superior to that of non-MSCs group (74% vs. 50%, *p* = 0.034). As pulmonary infection accounted for 67% and 70% of deaths in the two groups, respectively, the improvement in survival rate should be attributed to the treatment of pneumonia by mesenchymal stem cells. This therapeutic effect may mainly come from two characteristics of mesenchymal stem cells—immunomodulatory and tissue repair. After systemic administration, MSCs initially remain in the lungs, then “homing” and are retained in the injured or inflamed area [23]. Tracking studies using radioactive or fluorescent labeled MSCs have shown that MSCs stay in the pulmonary vascular bed for several days, and most cells are cleared within 24–48 h, which may be prolonged in damaged lungs. At the site of injury, MSCs secret soluble mediators (including anti-inflammatory cytokines, antimicrobial peptides, and vascular growth factors) and extracellular vesicles, and/or transfer mitochondria and other organelles to target immune cells (white blood cells - monocytes, macrophages, lymphocytes) and structural cells (including endothelial cells, epithelial cells, and smooth muscle cells) [24]. A recent study [25] investigated the mechanism of mesenchymal stem cell-derived exosomes in cytomegalovirus pneumonia. It was found that they inhibit the NF-κB/NLRP3 inflammatory signaling pathway, thereby modulating the excessive immune response and promoting the transformation of macrophages from the M1 to the M2 phenotype. This leads to alleviated lung inflammation, reduced tissue damage, and suppressed pulmonary fibrosis. Additionally, the immune system response of the recipient to dead or dying MSCs may also benefit lung injury [26].

MSCs contributed to the resolution of pneumonia, although this did not reach statistical significance. The reasons for the statistically negative results may include: (i) According to the retrospective study by Qu et al. [16], MSCs demonstrate significant efficacy in severely ill patients, whereas mild cases can recover with or without MSCs therapy. The inclusion of mild cases diluted the apparent difference in cure rates between the two groups, but the small sample size of this study precluded stratified analysis based on disease severity. (ii) The treatment course of MSCs was insufficient. Some patients received MSCs infusions during their initial hospitalization for CMV pneumonia and improved. However, after discharge, they experienced worsening pneumonia outside the hospital and did not receive timely subsequent MSCs treatment, indicating that a complete course of MSCs therapy is necessary for recovery. In this study, the maximum number of MSCs infusions was four, corresponding to a four-week course, shorter than the duration needed for pneumonia recovery. Increasing the infusion frequency could potentially enhance therapeutic efficacy.

Given that MSCs have been widely used in graft-versus-host disease after HSCT, MSCs may improve patient outcomes by alleviating or preventing acute GVHD. We further investigated the impact of GVHD on patient outcomes in our study. No significant difference was observed in the incidence of GVHD between the two groups (8/24 vs. 8/19, *P* = 0.555). Analysis of all 43 patients showed that the occurrence of GVHD did not significantly affect overall survival (*P* = 0.187). We postulate that this may be attributed to the overall low severity of GVHD observed in the enrolled patients. Each group contained only two patients with grade 2 acute GVHD; all other cases were grade 1. Such mild forms of GVHD may not exert a substantial impact on survival in either group [27]. Moreover, it is also plausible that GVHD control may have contributed to the improvement in CMV pneumonia.

Another limitation of this study is that the difference in the incidence of CMV viremia between the two groups (92% vs. 63%) may potentially raise doubts regarding the diagnostic accuracy. Although, under the criteria for probable diagnosis, there were no significant differences in the viral load of bronchoalveolar lavage fluid or imaging findings between the two groups, the difference in viremia rates could impact diagnostic accuracy and prognosis. This discrepancy may have been due to the relatively small sample size and the phenomenon that the CMV infection is more readily detected in bronchoalveolar lavage fluid than in plasma [18]. In a prior study, 11 of 61 patients were diagnosed with CMV pneumonia based solely on bronchoalveolar lavage fluid testing in the absence of viremia [28]. We investigated the impact of viremia on the pneumonia remission rate, time to remission, and overall survival among all 43 patients and found no significant differences, with P values of 0.398, 0.158, and 0.842, respectively.

A concern regarding the treatment of CMV pneumonia with MSCs is that the immunosuppressive effects of MSCs may exacerbate CMV replication and invasion. However, research by Helen Karlsson et al. [29] demonstrates that although MSCs exert a strong immunosuppressive effect on allogeneic reactive T cells, their impact on T cell responses to CMV is minimal. This suggests that the immunological function of CMV-specific T cells can be preserved following MSCs infusion. Additionally, according to the immunopathogenesis hypothesis of CMV infection (which posits that in the immune response to CMV, CD3 + T cells may exacerbate lesions as a side effect, with CD4 + T cells also being implicated [30]), the immunomodulatory effects of MSCs may help alleviate the clinical symptoms of CMV pneumonia and prevent death due to severe pneumonia. Furthermore, no adverse reactions associated with MSCs infusion were observed in this study, confirming the safety of MSCs.

## Conclusion

The high mortality rate of CMV pneumonia following allo-HSCT leads to poor patient outcomes. MSCs can improve the overall survival of pneumonia patients. The cure rate and time to cure of pneumonia were not improved statistically. Extending the infusion treatment course could potentially improve patient outcomes. Currently, there is a lack of prospective studies on the adjunctive treatment of pneumonia with MSCs after HSCT, which warrants further investigation in the future.

## Data Availability

The data analyzed in the current study are available from the corresponding author on reasonable request.
